# On the Importance of Electron Diffusion in a Bulk-Matter Test of the Pauli Exclusion Principle

**DOI:** 10.3390/e20070515

**Published:** 2018-07-09

**Authors:** Edoardo Milotti, Sergio Bartalucci, Sergio Bertolucci, Massimiliano Bazzi, Mario Bragadireanu, Michael Cargnelli, Alberto Clozza, Catalina Curceanu, Luca De Paolis, Jean-Pierre Egger, Carlo Guaraldo, Mihail Iliescu, Matthias Laubenstein, Johann Marton, Marco Miliucci, Andreas Pichler, Dorel Pietreanu, Kristian Piscicchia, Alessandro Scordo, Hexi Shi, Diana Laura Sirghi, Florin Sirghi, Laura Sperandio, Oton Vázquez Doce, Eberhard Widmann, Johann Zmeskal

**Affiliations:** 1 Dipartimento di Fisica, Università di Trieste and INFN-Sezione di Trieste, 34127 Trieste, Italy; 2 Laboratori Nazionali di Frascati, Istituto Nazionale di Fisica Nucleare, 00044 Frascati, Italy; 3 Dipartimento di Fisica e Astronomia, Università di Bologna, 40126 Bologna, Italy; 4 IFIN-HH, Institutul National pentru Fizica si Inginerie Nucleara Horia Hulubei, 077125 Mâgurele, Romania; 5 Stefan Meyer Institute for Subatomic Physics, Austrian Academy of Sciences, 1090 Vienna, Austria; 6 Centro Fermi, Museo Storico della Fisica e Centro Studi e Ricerche “Enrico Fermi”, 00184 Roma, Italy; 7 Institut de Physique, Université de Neuchâtel, 2000 Neuchâtel, Switzerland; 8 Laboratori Nazionali del Gran Sasso, Istituto Nazionale di Fisica Nucleare, 67100 Assergi, Italy; 9 Institut für Hochenergiephysik der Österreichischen Akademie der Wissenschaften, 1050 Vienna, Austria; 10 Excellence Cluster Universe, Technische Universität München, 85748 Garching , Germany

**Keywords:** Pauli exclusion principle, X-rays, diffusion processes, fundamental symmetries

## Abstract

The VIolation of Pauli (VIP) experiment (and its upgraded version, VIP-2) uses the Ramberg and Snow (RS) method (Phys. Lett. B **1990**, 238, 438) to search for violations of the Pauli exclusion principle in the Gran Sasso underground laboratory. The RS method consists of feeding a copper conductor with a high direct current, so that the large number of newly-injected conduction electrons can interact with the copper atoms and possibly cascade electromagnetically to an already occupied atomic ground state if their wavefunction has the wrong symmetry with respect to the atomic electrons, emitting characteristic X-rays as they do so. In their original data analysis, RS considered a very simple path for each electron, which is sure to return a bound, albeit a very weak one, because it ignores the meandering random walks of the electrons as they move from the entrance to the exit of the copper sample. These complex walks bring the electrons close to many more atoms than in the RS calculation. Here, we consider the full description of these walks and show that this leads to a nontrivial and nonlinear X-ray emission rate. Finally, we obtain an improved bound, which sets much tighter constraints on the violation of the Pauli exclusion principle for electrons.

## 1. Introduction

In 1990, Erik Ramberg and George A. Snow (RS) were the first to carry out a very clean experiment to test the validity of the Pauli exclusion principle [1], a cornerstone of modern physics [2]. In the RS experiment, electrons were forced to flow in a copper strip (the “target”) by circulating a direct current (DC), and it was assumed that if any of these electrons approached an atom where another electron had the “wrong” pairing with it, it could undergo radiative capture and emit a characteristic X-ray as it settled into an anomalous atomic ground state.

RS estimated a lower bound for the number of the emitted X-rays $N_{X}$ as follows:

(1) $$N_{X}\geq \frac{1}{2}\beta^{2}\times N_{\mathrm{new}}\times \frac{1}{10}\times N_{\min}\times \left(\mathrm{geometric}\;\mathrm{factor}\right),$$

where $\beta^{2}/2$ is the Pauli-violating probability, $N_{\mathrm{new}}$ is the total number of “new” conduction electrons injected into the system, the factor 1/10 is an estimate of the capture probability (per scattering) into the 2P state, $N_{\min}$ is the minimum number of electron-atom scatterings as electrons flow in the target and the “geometric factor” takes into account both the solid angle covered by the detector and the X-ray absorption in the copper strip. Since the number of new electrons depends on the current *I*, $N_{\mathrm{new}}=\Sigma I\Delta t_{M}/e$ and using the estimate $N_{\min}>L/\mu$ where *L* is the target length, $\Delta t_{M}$ is the total measurement time and μ is the scattering length for conduction electrons in the copper strip, RS found:

(2) $$N_{X}\geq \beta^{2}\frac{L\Sigma I\Delta t_{M}}{e\mu }\times \frac{1}{20}\times \left(\mathrm{geometric}\;\mathrm{factor}\right).$$

We expect the number of non-Paulian transitions, and correspondingly the number of emitted X-rays, to be very low and to find a value of β very close to zero. In the real world, experiments operate with non-negligible environmental X-ray backgrounds, and by comparing the number of measured X-rays with and without current in the target one finds an upper bound for $\beta^{2}/2$, both in the RS experiment [1] and in improved versions of the same experimental setup, such as VIolation of Pauli experiment (VIP) [3] and VIP-2 [4,5]. A first and rather obvious remark is that the minimum number of scatterings L/μ is far from being a realistic estimate of the actual number of scatterings, which must be much larger because electrons diffuse through the metal and perform complex random walks. Moreover, in addition to diffusion, there is also transport due to the potential difference in the target, which relates directly to the current and must be properly addressed to find the best estimate of the number of scatterings. However, if one tackles this problem in a naive way, a seemingly absurd situation soon arises. First, we note that the average transit time under the detector is $\Delta t_{T}=L/v_{d}$, where $v_{d}$ is the drift speed due to the potential difference *V* over the distance *L*. Then, we recall that:

(3) $$v_{d}=\frac{e\tau }{m_{e}L}V,$$

where 1/τ is the individual electron’s scattering rate, which is related to the mean free path and to the Fermi velocity, $1/\tau =v_{F}/\mu$, and:

(4) $$V=RI=\rho \frac{L}{A}I,$$

where $\rho =m_{e}/ne^{2}\tau$ is the conductivity and *n* is the (numeric) free (conduction band) electron density. Then, we find:

(5) $$v_{d}=\frac{e\tau }{m_{e}L}\frac{m_{e}}{ne^{2}\tau }\frac{L}{A}I=\frac{1}{ne}\frac{I}{A},$$

where *A* is the cross-section of the conductor. Each electron contributes on average $\Delta t_{T}/\tau$ scatterings, and since I/e electrons are introduced per unit time, there are $N_{\mathrm{scatt}}=\Delta t_{T}I/e\tau$ scatterings per unit time. Therefore:

(6) $$N_{\mathrm{scatt}}=\frac{\Delta t_{T}I}{e\tau }=\frac{L}{e\tau v_{d}}I=\frac{nLA}{\tau }=\mathrm{electrons}\;\mathrm{in}\;\mathrm{the}\;\mathrm{volume}\;\mathrm{below}\;\mathrm{the}\;\mathrm{detector}\times \mathrm{scattering}\;\mathrm{rate},$$

and we find that the average total number of scatterings $N_{\mathrm{scatt}}\times \Delta t_{M}$ in the measurement time $\Delta t_{M}$ does not depend on current . This result contradicts the RS analysis, and it is somewhat paradoxical since it seems that a more refined analysis of the experiment kills all efforts to use this scheme where we turn the current on and off. This begs for a deeper explanation.

In the next section, we solve the apparent paradox, with specific reference to the VIP and VIP-2 experiments (see [3,4,5] for a detailed description of these experiments).

## 2. Mathematical Model

In this section, we start by recalling some basic facts about electron conduction in metals, and next, we study a one-dimensional model of transport with diffusion that solves the paradox of the apparent equivalence between the current on and off states. We consider conduction in a rectangular strip, and whenever equations are translated into numbers, we refer to the VIP [3] and VIP-2 [4,5] experiments’ specifications listed in Table 1.

### 2.1. Quick Refresher of Some Basic Concepts of Electron Conduction in Metals

From the standard theory of electron conduction in metals, we know that the electron density *n* at 0 K is:

(7) $$n_{0}K=\frac{8\sqrt{2}\pi m_{e}^{3/2}}{h^{3}}\left(\frac{2}{3}E_{F}^{3/2}\right)\approx 6.846\times 10^{27}{\mathrm{eV}}^{-3/2}m^{-3}\left(\frac{2}{3}E_{F}^{3/2}\right).$$

In particular, the Fermi energy of copper is 7 eV, and therefore:

$$n_{0}K,\mathrm{Cu}\approx 8.46\times 10^{28}\;m^{-3}.$$

It is also well known that this value evaluated a 0 K is almost unchanged at room temperature. The average electron speed is, to a good approximation, determined by the Fermi energy:

(8) $$v_{F}\approx \frac{3}{4}\sqrt{\frac{2E_{F}}{m_{e}}}\approx 1.18\times 10^{6}m/s.$$

where the 3/4 factor comes from the integration of the approximately uniform Fermi–Dirac distribution at room temperature in momentum space. The mean free path μ is related to the conductivity σ and average electron speed by the equation:

(9) $$\mu \approx \frac{m_{e}v_{F}}{n_{0}K\;e^{2}}\sigma ,$$

so that the average collision frequency is:

(10) $$\frac{1}{\tau }\approx \frac{v_{F}}{\mu }\approx \frac{n_{0}K\;e^{2}}{m_{e}\sigma },$$

and for copper, which has a conductivity of $\sigma \approx 5.9\times 10^{7}\Omega^{-1}$m${}^{-1}$, we find μ≈39nm and $\tau \approx 2.5\times 10^{-14}\;s$.

The drift speed is given by the equation:

(11) $$v_{d}=\frac{eE\tau }{m_{e}}\approx \frac{1}{n_{0}K\;ezw}\bar{I},$$

where *w* is the metal strip width and *z* is its thickness. Here, the coefficient is ${\left(nezw\right)}^{-1}\approx 5.23\;\times \;10^{-6}$ m/C (VIP) or $7.38\times 10^{-4}$ m/C (VIP-2), so that with a 40-A current (VIP), the drift speed is about 0.21 mm/s, and with a 100-A current (VIP-2), the drift speed is about 7.4 mm/s. From this, we can compute the traversal time $\Delta t_{T}$ under the detector of size $L_{T}$

(12) $$\Delta t_{T}=\frac{nezwL_{T}}{\bar{I}},$$

which is about 420 s in VIP and 10 s in VIP-2.

Finally, we recall the Einstein relation for the diffusion constant of charged particles with electric mobility σ/ne at temperature *T*:

(13) $$D\approx \frac{\sigma k_{B}T}{n\;e^{2}},$$

and for electrons in copper, this evaluates to $D_{\mathrm{Cu}}\approx 1.1\times 10^{-4}$ m${}^{2}$/s at room temperature (300 K). Notice that in the same traversal time given above, we find that the root-mean-square (RMS) distance traveled in any one direction by diffusion is $r_{\mathrm{RMS}}=\sqrt{D\Delta t_{T}}$, which is $r_{\mathrm{RMS}}\approx 20$ cm in VIP and 3 cm in VIP-2.

### 2.2. A Simple 1D Diffusion-Transport Model

Here, we consider a simple 1D diffusion-transport model. It is well known that after injection, a charge drifts and scatters (it undergoes transport and diffusion) so that the probability density of finding it at position x=x(t) at time *t*, given the starting position x(0)=0, is:

(14) $$p\left(x|t\right)=\frac{1}{\sqrt{2\pi Dt}}\exp\left\{-\frac{{\left[x-v_{d}t\right]}^{2}}{2Dt}\right\},$$

which is the Green’s function of the diffusion equation for non-interacting random walks; see, e.g., [6]. This means that the probability of actually finding one such electron in the target at time *t* is given by the integral:

(15) $$P\left(t\right)=\int_{0}^{L}p\left(x|t\right)dx=\int_{0}^{L}\frac{1}{\sqrt{2\pi Dt}}\exp\left(-\frac{{\left(x-v_{d}t\right)}^{2}}{2Dt}\right)dx,$$

where the target spans the *x*-interval (0,L). The integral (15) can be evaluated using the usual definition of the error function:

(16) $$\mathrm{erf}\left(z\right)=\frac{2}{\sqrt{\pi }}\int_{0}^{z}e^{-t^{2}}dt,$$

by setting $z=\left(x-v_{d}t\right)/\sqrt{2Dt}$ so that $x=\sqrt{2Dt}\;z+v_{d}t$ and $dx=\sqrt{2Dt}\;dz$, the integral becomes:

(17) $$\begin{array}{cc} \int_{0}^{L}\exp\left(-\frac{{\left(x-v_{d}t\right)}^{2}}{2Dt}\right)dx=\sqrt{2Dt} & \int_{-v_{d}t/\sqrt{2Dt}}^{\left(L-v_{d}t\right)/\sqrt{2Dt}}e^{-z^{2}}dz\\ & =\sqrt{\frac{\pi Dt}{2}}\left[\mathrm{erf}\left(\left(L-v_{d}t\right)/\sqrt{2Dt}\right)-\mathrm{erf}\left(-v_{d}t/\sqrt{2Dt}\right)\right], \end{array}$$

and finally:

(18) $$P\left(t\right)=\frac{1}{2}\left[\mathrm{erf}\left(\left(L-v_{d}t\right)/\sqrt{2Dt}\right)-\mathrm{erf}\left(-v_{d}t/\sqrt{2Dt}\right)\right].$$

Then, the number of scatterings that is observed in the time interval (t,t+dt) is just P(t)dt/τ, and therefore, the total number of scatterings observed for this single electron is:

(19) $$S\left(\Delta t_{M},I\right)=\frac{1}{\tau }\int_{0}^{\Delta t_{M}}P\left(t\right)dt,$$

which depends both on the time interval $\Delta t_{M}$ and on the current, by way of the dependence $v_{d}\left(I\right)=I/newz$. According to the RS analysis, in a fraction $\beta^{2}/20$ of all scatterings, electrons can be captured, so that the number of produced X-rays is:

(20) $$N_{X}=\frac{\beta^{2}}{20}S\left(\Delta t_{M},I\right)=\frac{\beta^{2}}{20\tau }\int_{0}^{\Delta t_{M}}P\left(t\right)dt.$$

The exact evaluation of the integral in Equation (20) can only be done in special cases, in particular in the case:

(21) $$\begin{array}{cc} S_{\infty }\left(I\right) & =S(\infty ,I)\\ & =\frac{1}{\tau }\int_{0}^{\infty }P\left(t\right)dt=\frac{1}{2\tau }\int_{0}^{\infty }\left[\mathrm{erf}\left(\left(L-v_{d}t\right)/\sqrt{2Dt}\right)-\mathrm{erf}\left(-v_{d}t/\sqrt{2Dt}\right)\right]dt\\ & =\frac{1}{\tau }\int_{0}^{\infty }dt\int_{0}^{L}\frac{1}{\sqrt{2\pi Dt}}\exp\left(-\frac{{\left(x-v_{d}t\right)}^{2}}{2Dt}\right)dx. \end{array}$$

We use the special integral:

(22) $$\int_{0}^{\infty }dt\int_{0}^{1}\frac{1}{\sqrt{2\pi Dt}}\exp\left(-\frac{{\left(x-t\right)}^{2}}{2Dt}\right)dx=1,$$

and using the substitutions x=Ls and $y=t(v_{d}/L)$, we find:

(23) $$S_{\infty }\left(I\right)=\frac{L}{v_{d}\tau }=\frac{\Delta t_{T}}{\tau },$$

and this result is the same as that found in the previous section with the very naive model, so that even in this better-defined context, there is no dependence on current. However, the model also underscores the importance of the time development of the signal.

**Figure 1:**
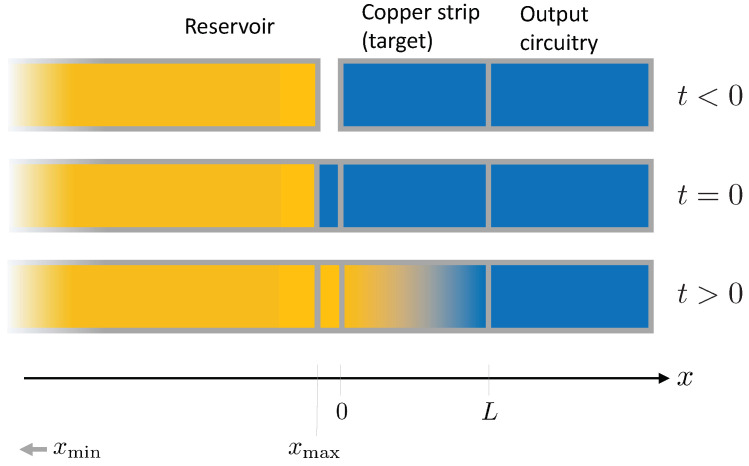
Schematics of “new” electron injection. Initially (t<0), the target is separate from the large reservoir of “new” electrons, which in this simple scheme extends from a faraway starting coordinate $x_{\min}$ to $x_{\max}$. At time t=0, the reservoir is put in contact with the target (for instance, with a switch that contributes a small amount of copper), and electrons can flow (t>0) from the source towards the output circuitry. Light orange marks a high density of “new” electrons, while blue marks a low electron density. The “output circuitry” stands for the rest of the circuit, where electrons eventually drift.

To clearly define timing, we need to modify the usual RS experimental scheme. Initially, the copper strip (the target) is separated from the reservoir of “new” electrons that have never been close to the atoms in the target. At time t=0, the reservoir is put in contact with the target, and electrons can flow from the source towards the output circuitry (t>0). As electrons traverse the target, if there is a violation of the Pauli exclusion principle, they can be captured by the atoms and cascade electromagnetically to the fully-occupied ground level. This whole scheme is shown pictorially in Figure 1.

We can assume that the “new” electrons are uniformly distributed in the reservoir. This means that the probability density function of the position *x* of an individual electron at time *t* is:

(24) $$\begin{array}{cc} p(x,t|x_{\min},x_{\max},v_{d}) & =\int_{x_{\min}}^{x_{\max}}p\left(x,t|x_{0},v_{d}\right)p\left(x_{0}\right)dx_{0}\\ & =\frac{1}{x_{\max}-x_{\min}}\int_{x_{\min}}^{x_{\max}}\frac{1}{\sqrt{2\pi Dt}}\exp\left(-\frac{{\left(x-x_{0}-v_{d}t\right)}^{2}}{2Dt}\right)dx_{0}\\ & =\frac{1}{2(x_{\max}-x_{\min})}\left[\mathrm{erf}\left(\frac{x-x_{\min}-v_{d}t}{\sqrt{2Dt}}\right)-\mathrm{erf}\left(\frac{x-x_{\max}-v_{d}t}{\sqrt{2Dt}}\right)\right] \end{array}$$

where $x_{0}$ is the initial position of the electron, $x\left(0\right)=x_{0}$, the length $x_{\max}-x_{\min}$ is the total length of the reservoir (here, we assume for simplicity a reservoir that has the same cross-section as the target and such that after being connected to the target, it starts at position $x_{\min}$ and ends at position $x_{\max}$) and $p\left(x_{0}\right)={\left(x_{\max}-x_{\min}\right)}^{-1}$ is the uniform probability density function for the initial position of the electron.

**Figure 2:**
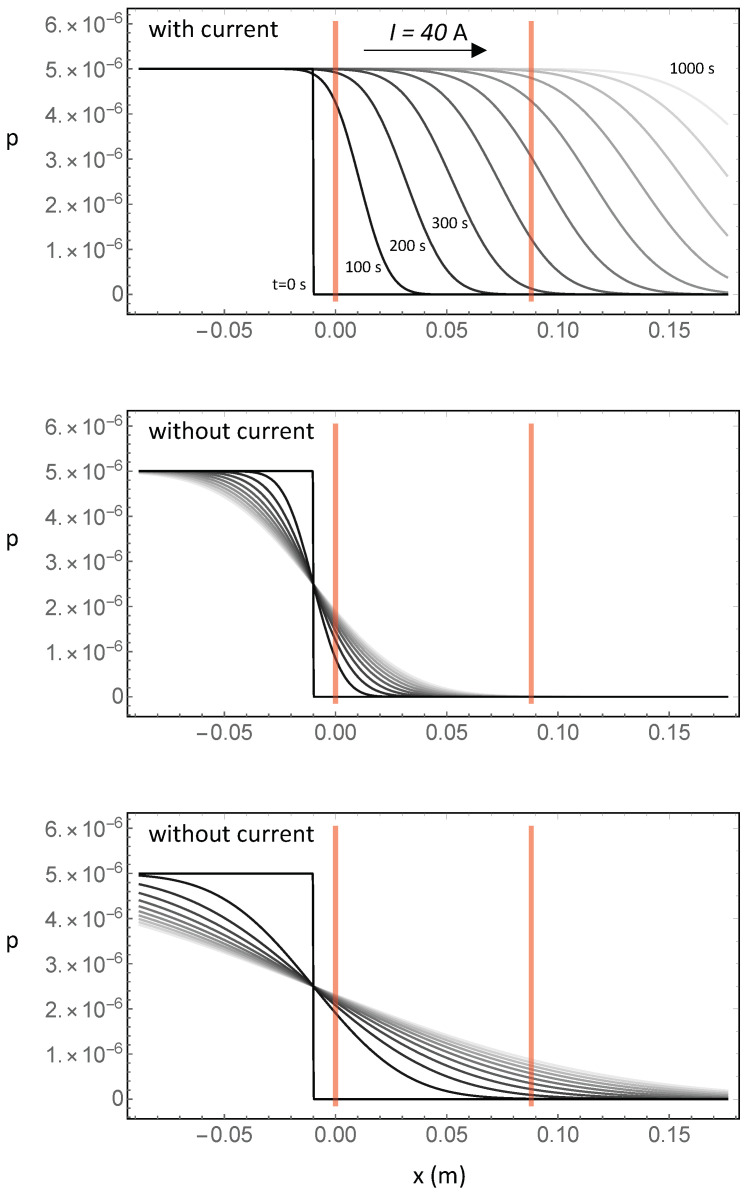
Probability density function (pdf) for finding a single “new” electron with a reservoir that has a volume about $2.3\times 10^{6}$-times larger than the target (corresponding to about 0.25 m${}^{3}$ of copper, with a mass of about 2.2 metric tons), with and without current. The red vertical bars mark the start and the end of the target; the reservoir is attached with a 1-cm connector to the target, and to obtain clearer figures, the diffusion constant has a value that is 100-times smaller than the true one. **Top panel:** pdf with a 40-A current at times 0 s–1000 s after connecting the external reservoir. The curves are taken at 100-s intervals and are labeled with the corresponding times (except the middle ones), as well as with different gray levels. Initially, the pdf is a uniform distribution with a sharp step at the boundaries of the reservoir; a large part of it is in the reservoir and is not shown. Eventually, in this case after about 1000 s, the right-propagating step of the pdf moves beyond the target, and the distribution inside the target is uniform. **Middle panel:** pdf without current. The curves are labeled with gray levels only. **Bottom panel:** pdf without current over a longer time span, up to 10,000 s, with 1000-s intervals. This is a limiting case that corresponds to a reservoir that is connected to the target, which would however never occur in practice because the no-current case corresponds to a disconnected reservoir (and therefore, to no newly-injected electrons).

Figure 2 illustrates what happens in two different situations—without current and with current—when we consider the experimental arrangement of Figure 1: it is quite clear that the signal development, which is associated with the probability of finding a “new” electron in the target, is very different in the two cases. With current, electrons from the source travel fast and soon fill the whole target, even though, in measurements without current, the electrons that diffuse from the reservoir into the target also produce a signal; however, this takes more time, and the “new electron” density is lower than in the case with the current.

The total number of “new” electrons associated with this reservoir is obviously $nA(x_{\max}-x_{\min})$, and the number of “new” electrons in any slice of thickness Δx at position *x* and time *t* is:

(25) $$nA\left(x_{\max}-x_{\min}\right)p\left(x,t|x_{\min},x_{\max},v_{d}\right)\Delta x=nA\Delta x\left[\mathrm{erf}\left(\frac{x-x_{\min}-v_{d}t}{\sqrt{2Dt}}\right)-\mathrm{erf}\left(\frac{x-x_{\max}-v_{d}t}{\sqrt{2Dt}}\right)\right].$$

Finally, the expected number of anomalous X-rays per unit time at time *t* is:

(26) $$\frac{\beta^{2}}{2}r\nu nA\int_{0}^{L}\left[\mathrm{erf}\left(\frac{x-x_{\min}-v_{d}t}{\sqrt{2Dt}}\right)-\mathrm{erf}\left(\frac{x-x_{\max}-v_{d}t}{\sqrt{2Dt}}\right)\right]dx,$$

where *r* is the radiative capture probability per scattering and ν is the scattering rate, and the total number of emitted X-rays during the data-taking time $\Delta t_{M}$ is:

(27) $$\frac{\beta^{2}}{2}r\nu nA\int_{0}^{\Delta t_{M}}\int_{0}^{L}\left[\mathrm{erf}\left(\frac{x-x_{\min}-v_{d}t}{\sqrt{2Dt}}\right)-\mathrm{erf}\left(\frac{x-x_{\max}-v_{d}t}{\sqrt{2Dt}}\right)\right]dx\;dt$$

(note that this equation does not take into account the geometric factor listed in Table 1).

**Figure 3:**
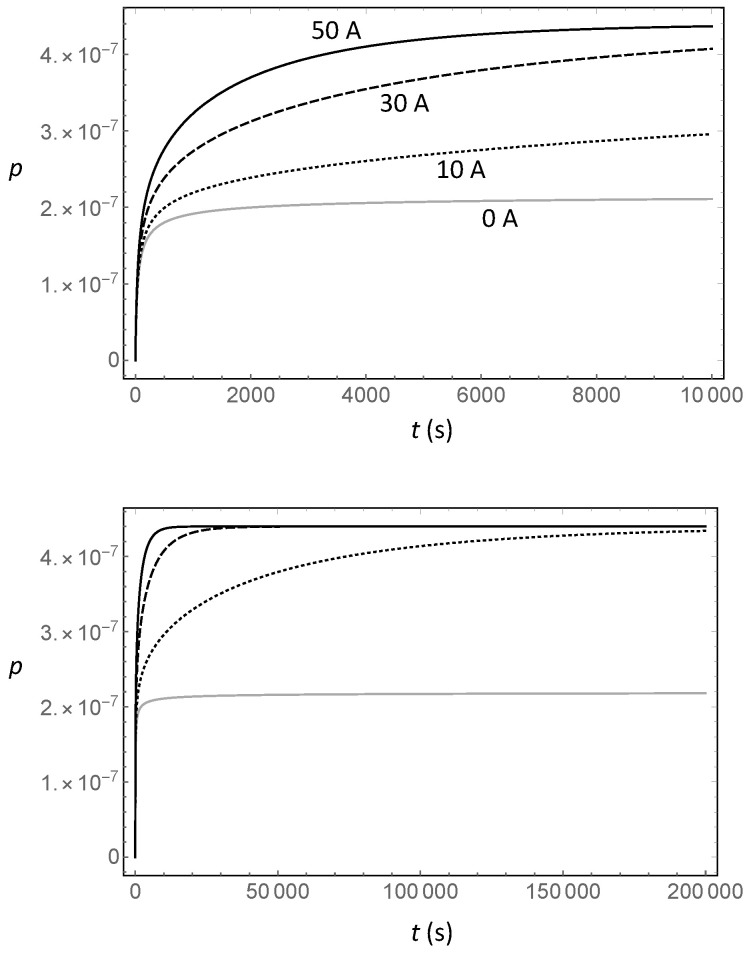
(**Top panel**) Short-time development of the integral (24), which is the single electron probability density and contains the time dependence in Equation (26), for different values of the current $\bar{I}$ and for the same reservoir size as Figure 2. (**Bottom panel**) Long-time development of the integral (24) for the same values of the current $\bar{I}$ shown in the top panel.

Figure 3 shows the time development of the X-ray signal (26) in different conditions: the asymptotic behavior is the same for different values of the current, while the short-time behavior is very different. The no-current case also yields a non-negligible probability, as diffusion spreads the “new” electrons in the target. This means that the “current off” state must correspond to a physically-disconnected reservoir in order to yield a null signal.

## 3. Discussion

In the previous section, we have considered the diffusion model without reassessing the numbers that are to be used to find the actual bound on the validity of the Pauli exclusion principle. In particular, we have postponed a discussion of the scattering frequency ν. If we were to replicate the RS idea that important scatterings are those that contribute to conduction, then ν=1/τ, and the total number of scatterings during the traversal time is $\Delta t_{T}/\tau$, which is about $1.3\times 10^{16}$ in VIP and $3\times 10^{14}$ in VIP-2. Using the original RS proposal (the straight path) would lead to about $2.2\times 10^{6}$ scatterings in VIP and to about $1.8\times 10^{6}$ scatterings in VIP-2. Thus, a proper consideration of the electrons’ paths leads to amplification by a factor of about $10^{10}$ in VIP and more than $10^{8}$ in VIP-2.

However, the problem with the scattering frequency ν is that the estimate provided by RS is not actually relevant to the test of the Pauli exclusion principle. Indeed, the scatterings that are considered in the RS paper are mostly electron-phonon scattering (in addition to other lattice irregularities like dopants, lattice dislocations, etc.) and have nothing to do with the actual electron capture process. Here, we replace these electron scatterings with the “close encounters” with individual atoms and give a rough estimate of their frequency starting from the electron wavelength $\lambda_{e}=h/m_{e}v\approx h/m_{e}v_{F}$. In Cu, this means $\lambda_{e}\approx 6.1\times 10^{-10}$ m. The radiative capture probability per close encounter can be estimated from the measured width of the naturally-occurring K${}_{\alpha }$ line complex Γ≈2.73 eV (weighted value for the whole K${}_{\alpha }$ complex; values taken from [7]) and from the transit time $\lambda_{e}/v_{F}\approx 5.3\times 10^{-16}$ s. A rough approximation for *r* yields $r\approx \left(\hbar /\Gamma \right)\times \left(\lambda_{e}/v_{F}\right)\approx 1.6>1$; this means that we can assume that every close encounter leads to a capture with a probability that is only limited by the Pauli violating probability $\beta^{2}/2$.

Now, using the computed electron wavelength and the electron density in copper, we find the average distance between close encounters with atoms, $\ell \approx 1/n\pi {\left(\lambda_{e}/2\right)}^{2}\approx 41$ pm, and the corresponding mean time between close encounters = $\ell /v_{F}\approx 3.5\times 10^{-17}$ s. This means that with a 40-A current and a mean traversal time of 420 s (as in VIP), there are on average at least $1.2\times 10^{19}$ close encounters, instead of the ≈$2.3\times 10^{6}$ scatterings that can be computed from the RS approach, based on a straight path below the detector. Then, the bound that is obtained using the viewpoint exposed in this paper is better than the bound found with the RS approach by a factor $5.2\times 10^{12}$. In the case of VIP-2 ($\bar{I}=100$ A) with a mean traversal time of about 10 s, there are on average at least $2.8\times 10^{17}$ close encounters instead of about $1.8\times 10^{6}$, and the bound improves by a factor $1.6\times 10^{11}$. If we take the more recent VIP-2 experiment [5], this translates into a bound on the violation parameter: $\beta^{2}/2<2.6\times 10^{-40}$.

The violation parameter can be understood in the context of non-relativistic quantum mechanics, either with a model of small violations like that of Ignatiev and Kuzmin [8] (see also [9,10,11]) or in the framework of electrons with a mixed symmetry state like in the paper by Rahal and Campa [12] and in non-relativistic quantum field theory with a model like the quons proposed by Greenberg [13]. In previous publications, we have considered our data either in the framework of the model of Ignatiev and Kuzmin or Greenberg’s quons: however, these models share common difficulties, highlighted in the past by Greenberg and Govorkov [14,15,16,17].

Rahal and Campa [12] introduced the violation parameter as a normalized count of the configurations of all the electrons in the universe where a given subset of electrons commute, so that the global electron-wave function has only an approximate antisymmetric character. Non-relativistic quantum mechanics allows such a mixed symmetry wavefunction, which is instead ruled out by the spin-statistics theorem [18]; therefore, within such an interpretation, an RS-like experiment is a direct test of the spin-statistics connection. It is interesting to remark that taking the estimate of the observable mass of the whole universe, $M\approx 1.6\times 10^{55}$ g [19] (similar to many other recent estimates), and assuming that it is nearly all composed of hydrogen atoms, we find that the total number of electrons in the observable universe is about $10^{79}$, and then, as a consequence, the bound from VIP-2 means that less than $10^{39}$ electron pairs in the universe can actually have the wrong symmetry pairing.

## 4. Conclusions

By analyzing the motion of the electrons in the context of a classical random walk, we have found that for long times, there is no difference in the rate of the anomalous X-rays produced by different DC currents flowing in the target of the VIP and VIP-2 experiments, while the time required to achieve this X-ray rate depends strongly on the current. The extreme case of the reservoir connected with no current produces a final X-ray rate that is just half of the case with current because there is no drift, and half of the electrons move into the target, while the other half move away from it (this result is strictly true only for an infinitely long reservoir).

We note that the results are robust: transport is effectively one-dimensional because of the target shape, and it determines the traversal time $\Delta t_{T}$; however, we obtain time τ from a calculation that is not influenced by dimensional considerations, and the final estimate of the amplification factors does not depend on the dimension.

We also found that a large current may be detrimental to the experiment because it reduces the actual number of interactions between electrons in the target as they move from the entrance to the exit. This leads to some important considerations. The first is that there should be an optimal value of the current, such that the X-ray rate reaches the stationary value soon enough, but is not so large that the electrons do not have time to wander around and interact with as many electrons as possible in the target. Secondly, the time dependence can potentially be used to the experiment’s advantage, as it modulates the X-ray rate. In this way, we could use powerful signal analysis techniques like those introduced in [20] to further reduce the effect of the (unmodulated) background.

Finally, we note that the present analysis of the random walk is mostly classical and that we plan to extend it to the quantum domain. All these considerations suggest far-reaching work that we shall develop in the near future.

## Figures and Tables

**Table 1:** Main specifications of the VIolation of Pauli (VIP) and VIP-2 experiments, relevant to this paper. Note that the “geometric factor” includes the X-ray absorption length λ, not discussed elsewhere in this paper, but considered, e.g., in [3,4,5].

	VIP	VIP-2
Target material	Cu	Cu
Copper target shape	Cylinder (45-mm radius)	Strip
Copper target length (*L*)	88 mm	71 mm
Copper target thickness (*z*)	50 μm	50 μm
Copper target width (*w*)	282.74 mm	20 mm
Target cross-section (zw)	$1.41\times 10^{-5}$ m${}^{2}$	$1\times 10^{-6}$ m${}^{2}$
Target volume (zwL)	$1.24406\times 10^{-6}$ m${}^{3}$	$7.1\times 10^{-8}$ m${}^{3}$
Detectors (multiplicity)	Pairs of rectangular Charge Coupled Devices (CCD) in octagonal arrangement about the target (8 pairs)	1 cm${}^{2}$× 3 (on one side of the Cu strip) × 2 (two sides)
Geometric factor (λ/z)×(Ω/4π)	0.01	0.018
